# Embryology of the Abdominal Wall and Associated Malformations—A Review

**DOI:** 10.3389/fsurg.2022.891896

**Published:** 2022-07-07

**Authors:** Elisabeth Pechriggl, Michael Blumer, R. Shane Tubbs, Łukasz Olewnik, Marko Konschake, René Fortélny, Hannes Stofferin, Hanne Rose Honis, Sara Quinones, Eva Maranillo, José Sanudo

**Affiliations:** ^1^ Institute of Clinical and Functional Anatomy, Medical University of Innsbruck (MUI), Innsbruck, Austria; ^2^ Department of Neurosurgery, Tulane Center for Clinical Neurosciences, Tulane University School of Medicine, New Orleans, LA, United States; ^3^ Department of Neurology, Tulane Center for Clinical Neurosciences, Tulane University School of Medicine, New Orleans, LA, United States; ^4^ Department of Anatomical Sciences, St. George’s University, St. George’s, Grenada, West Indies; ^5^ Department of Structural & Cellular Biology, Tulane University School of Medicine, New Orleans, LA, United States; ^6^ Department of Surgery, Tulane University School of Medicine, New Orleans, LA, United States; ^7^ Department of Neurosurgery and Ochsner Neuroscience Institute, Ochsner Health System, New Orleans, LA, United States; ^8^ University of Queensland, Brisbane, Australia; ^9^ Department of Anatomical Dissection and Donation, Medical University of Lodz, Lodz, Poland; ^10^ Department of General, Visceral, and Oncological Surgery, Wilhelminenspital, Vienna, Austria; ^11^ Department of Anatomy and Embryology, School of Medicine, Complutense University of Madrid, Madrid, Spain

**Keywords:** embryology, human, abdominal wall, congenital hernia, developmental cascade

## Abstract

In humans, the incidence of congenital defects of the intraembryonic celom and its associated structures has increased over recent decades. Surgical treatment of abdominal and diaphragmatic malformations resulting in congenital hernia requires deep knowledge of ventral body closure and the separation of the primary body cavities during embryogenesis. The correct development of both structures requires the coordinated and fine-tuned synergy of different anlagen, including a set of molecules governing those processes. They have mainly been investigated in a range of vertebrate species (e.g., mouse, birds, and fish), but studies of embryogenesis in humans are rather rare because samples are seldom available. Therefore, we have to deal with a large body of conflicting data concerning the formation of the abdominal wall and the etiology of diaphragmatic defects. This review summarizes the current state of knowledge and focuses on the histological and molecular events leading to the establishment of the abdominal and thoracic cavities in several vertebrate species. In chronological order, we start with the onset of gastrulation, continue with the establishment of the three-dimensional body shape, and end with the partition of body cavities. We also discuss well-known human etiologies.

## Contribution to the Field Statement

Malformations of the abdominal wall and the cavities have become increasingly frequent in everyday life of specialized surgeons, but our knowledge of the pathomechanisms are still incomplete.

To set adequate interventions deep and fundamental knowledge about the organogenesis is required. This review reflects the essential steps of body wall and cavities formation starting with the establishment of the bilaminar disc and its further course of establishment of the body axis and three-dimensional patterning of the embryo. Major key genes, their influence in these processes as well as of the result when they are interrupted by intrinsic and extrinsic factors are mentioned. Furthermore, the connex between individual steps in embryogenesis and associated malformation are highlighted with special focus on the current state of knowledge and with special reference to recent studies on the subject. Studies on animal models were also included, as they represent an intrinsically important link and basis for understanding human embryology, however, we also pointed out the differences with human development.

This review provides a solid basis of knowledge for interested professionals and summarizes histological and molecular events leading to the establishment of the abdominal and thoracic cavities in several vertebrate species.

## Introduction

In vertebrates, the body wall is composed of skin, muscles, and associated connective tissue. Its establishment requires successive, well-coordinated processes during embryogenesis. Because of stringent ethical standards we know little about the development of the human body wall, few histological and morphological data being available for researchers (1–4). Therefore, most of our knowledge derives from animal models, extensive research having been conducted on mice. In murine models, several molecular signaling pathways essential for the normal formation of abdominal wall components have been discovered. Based on these studies, human embryology has made tremendous progress in recent decades and several molecular processes during early development (eight weeks after fertilization) have been comprehensively described (5–7). However, this promising gain in knowledge has a downside if we consider the fundamental differences in development between mice and humans. The time frame of differentiation and maturation of organs differ significantly, making comparisons between the two species difficult (8). Furthermore, the timings of blastocyst formation and gastrulation clearly differ (8). Although mice and humans share many genes in common, striking differences are evident (9, 10). The mouse genome is about approximately 14% smaller than the human genome, and mice have twice as many nucleotide substitutions as humans, which is reflected not only in different body plans but also in gene expression and reproductive strategies (8, 11). Knockout mice can serve as excellent research tools for investigating human malformations, but it should be remembered that several of these mice do not develop into adults, making it difficult to determine the gene’s function in relation to human health.

The establishment of the two body cavities and the closure of the body wall require an orchestrated synergy of multiple developmental processes. If this fails to occur during embryogenesis, surgeons are confronted with severe anatomical malformations in newborns: congenital diaphragmatic hernia and body wall defects such as gastroschisis and omphalocele.

These defects of the abdominal cavity and wall development have become increasingly frequent in recent years but our knowledge of the physiological and pathophysiological processes leading to such defects is still very limited. According to current knowledge they are mostly caused by epigenetic factors; chromosomal aberrations often account for only a fraction. Future research should therefore focus among other things, on these environmental factors and their impact on the pathogenesis of abdominal malformations. For example in 1963, Duhamel proposed that teratogens could interrupt the lateral folding of the embryo and therefore cause the development of gastroschisis (12). However, this hypothesis fell into oblivion in recent decades, the scientific focus having been on chromosomal aberrations.

**Figure 1:**
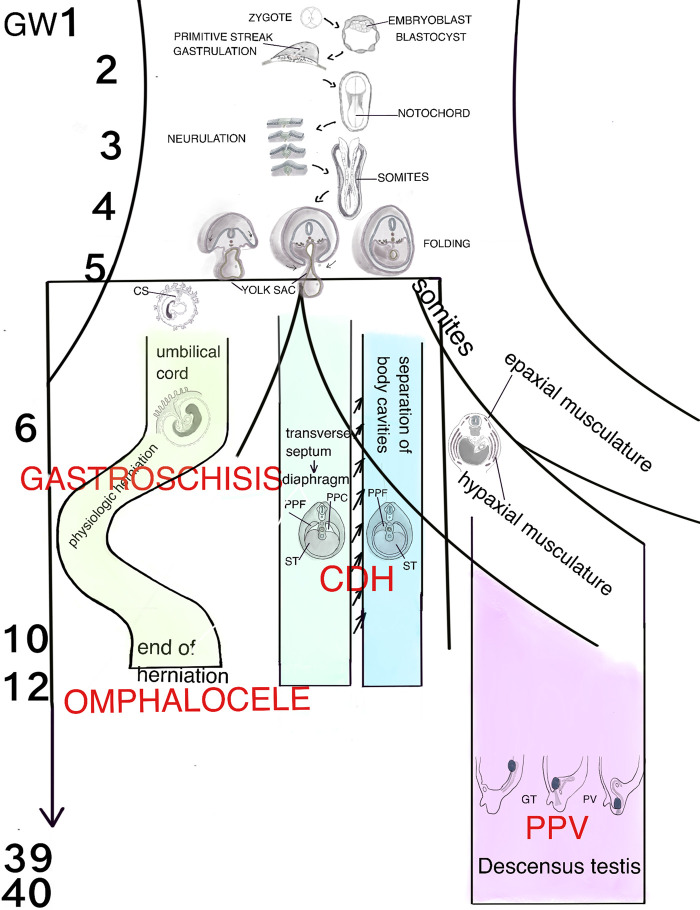
Schematic representation of the human organogenesis: Following fertilization the zygote transforms after cleavage and compaction into the blastocyst. At the end of the 2nd week the primitive streak appears on the surface of the embryo which is the first evidence of the beginning of gastrulation which ends with a trilaminar embryo. The notochord**,** a transient, rod-shaped structure induces neurulation and differentiation of the somites. Because of the rapid growth of the somites and the lateral plate mesoderm (LMP) the process of folding is initiated. The yolk sac is thereby incorporated into the embryonic body and the common body cavity is formed. The formation of the umbilical cord begins around week 3 with formation of the connecting stalk (CS). Approximately at week 7 the UC is fully established and is able to take over the metabolic functions. Physiologic herniation, due to the rapid growth of the intestine starts at week 6 and is terminated in the tenth week by its withdrawal into the embryonic body. If this does not take place, it comes to the formation of a omphalocele. If the amnion ruptures in the eighth to tenth week, gastroschisis results. The transverse septum (ST), which is located behind the base of the pericardial cavity separates the common body cavity incompletely since the pleuroperitoneal canals (PPC) on both sides are continuous between the two cavities. Due to the growth of the embryo, fusion of the pleuroperitoneal folds (PPF) occurs which leads to an occlusion of the canals. Morphogenetic defects of the PPF s subsequently prevent proper establishment of the costal muscles and its surrounding connective tissue with deficiencies in the diaphragmatic barrier. Differentiation of the inguinal canal closely is connected to differentiation of the gonads and their migration into the extracorporal scrotum together with the processus vaginalis, which is guided by the gubernaculum testis (GT). The IC acquires its adult morphology during the fetal period, due to the continuous growth of the abdominal muscles and wall, with the accompanying displacement of the inguinal rings. Failure in obliteration of the vaginal process will result in a patent processus vaginalis (PPV).

To understand the consequences of these extrinsic and intrinsic interrupters for differentiating organ systems, one must also understand the temporal, spatial, and morphogenetic sequence of organogenesis. The emerging field of genomic hybridization and third generation sequencing, and transcriptome analysis as well as synthetic human embryology using human pluripotent stem cells and organoids enable researchers to study disease mechanism as well as gain knowledge about human development (13). In the following chapters we recapitulate human organogenesis with special emphasize on morphological and structural differentiation. If there are significant animal models to add molecular aspects to the knowledge of organogenesis and its disruptions, they will be mentioned and discussed here (Figure 1).

## Recapitulation of Human Organogenesis

### Gastrulation

**Figure 2:**
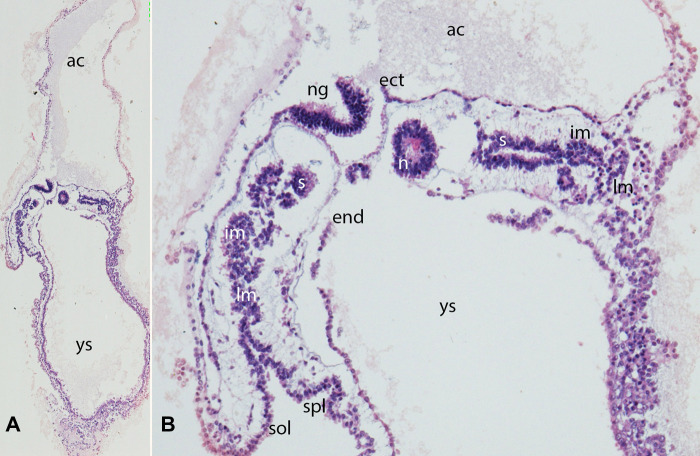
(**A,B**) Embryo GV-2 (6 somites). Stage 10 (4–12 somites; 2–3.5 mm; 22 days). Collection Orts LLorca (Complutense University of Madrid) Photos provided by Prof. J.F. Rodriguez-Vazquez. Oblique axial sections of the cephalic segment. (**A**) (4X) and (**B**) (10X). Staining: H-E. The trilaminar disc is located between the amniotic (ac) and yolk cavities (yc). In the ectoderm (ect) appears the neural groove (ng). The endoderm (end) is broken in segments. The mesoderm is divided into three segments: paraxial or somites (s), intermediate (im) and lateral (lm) mesoderm. The lateral mesoderm is split into the visceral/splanchnopleural (spl) and parietal/somatopleural (sol) layers. The notochord (n) appears as a cylindric structure with a cavity inside

**Figure 3:**
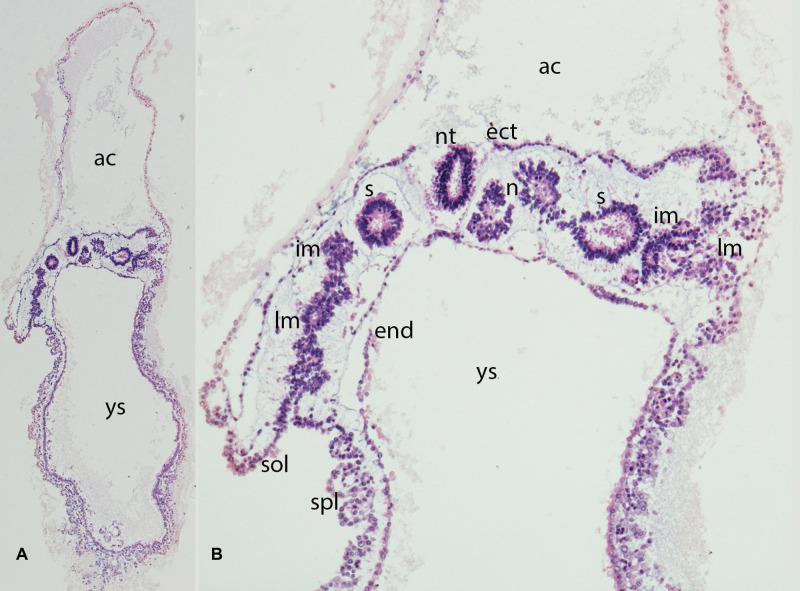
(**A,B**) Embryo GV-2 (6 somites). Stage 10 (4–12 somites; 2–3.5 mm; 22 days). Collection Orts LLorca (Complutense University of Madrid) Photos provided by Prof. J.F. Rodriguez-Vazquez. Oblique axial sections of the future thoracic segment. (**A**) (4X) and (**B**) (10X). Staining: H-E. The trilaminar disc is located between the amniotic (ac) and yolk cavities (yc). In the ectoderm (ect) appears the neural tube (nt). The endoderm (end) is broken in segments. The mesoderm is divided into three segments: paraxial or somites (s), intermediate (im) and lateral (lm) mesoderm. The lateral mesoderm is split into the visceral/splacnopleural (spl) and parietal/somatopleural (sol) layers. The notochord (n) appears as a double structure due to the obliquity of the section.

After compaction, the morula transforms into the blastocyst and loses its totipotency. The embryoblast arranges itself from the inner cell mass; the outer cell layer becomes the trophoblast, which provides nutrients and ensures implantation into the endometrium. Two distinct cell types are differentiated: the epiblast adjacent to the amniotic cavity, and the hypoblast facing the blastocyst cavity. The amnioblasts are located adjacent to the trophoblast and are continuous with the epiblast. The radially oriented cells from the epiblast are now surrounded by the amniotic cavity. The hypoblast or visceral endodermal cells delaminate from these epiblast cells, become separated from them by a basal lamina, and subsequently line the secondary yolk sac. The dorsoventral body axis is now determined as these two cell layers, the epiblast and hypoblast, emerge. During gastrulation, the two-dimensional shape transforms into a three-dimensional and trilaminar disc, which finally comprises the three germ layers (Figures 2, 3) (8, 14). At the end of the second week the primitive streak, which ends in the primitive knot, appears on the surface of the ectodermal layer and grows towards to the prechordal plate, a group of enlarged hypoblastic cells underlying the epiblast (6, 7). The primitive streak is the first evidence of the beginning of gastrulation and the subsequent establishment of the three germ layers. It is a region of pluripotent epiblast epithelium limited by the primitive node, which ingresses and undergoes epithelial to mesenchymal transition (EMT) (15). The primitive streak also indicates bilateral symmetry, with clear distinctions of right from left and caudal from cranial. This is evolutionarily conserved by gradients of morphogens that are regulated by positive and negative feedback mechanisms. Retinoid acid is one of the best studied morphogens, crucial for the spatial patterning of the mesodermal anlagen (16). Mouse models have revealed that Zic3 a member of the zinc finger protein family is critical for right-left differentiation, among other things (17, 18).

Epiblast cells migrate from the surface towards the streak, round off, and subsequently leave the epithelial cell complex to establish the mesodermal layer in the cleavage space between epiblast and hypoblast. Due to the detachment of the cell complex and the loss of apicobasal polarity, the cells lose their epithelial properties and reassume a mesenchymal character with the ability to migrate. This process is referred to as “epithelial to mesenchymal transition” or ingression (EMT). The morphological features for determining the body axes and planes are now defined. The mesoderm created by gastrulation is now divided into three functionally distinct sections: paraxial, intermediate, and lateral plate. The primitive segments, the so-called somites, originate in the paraxial mesoderm (Figures 2, 3).

### The Notochord

By the end of the third week, the embryo has acquired a typically disc-like shape composed of three germ layers; it is positioned between the amniotic cavity dorsally and the yolk sac ventrally. At week four after fertilization, the epiblast cells that invaginated the primitive knot migrate cranially towards the prechordal plate to form the notochord, a conserved, mesodermal, rod-shaped, and transient structure that stretches along the entire axial anterior-posterior midline (Figures 2, 3) (19, 20). This structure is essential for the neural and endodermal patterning of e.g., the intestine, liver, and lungs. In vertebrates signaling molecules such as hedgehog proteins secreted by the notochord play key roles regarding differentiation and growth of the surrounding tissues (21, 22).

The notochord is also the major player in establishing the anterior and posterior body axis and the folding process in vertebrate embryos (22–26). At the junction between the notochord and the primitive cord, the neurenteric canal forms as an epithelial depression and provides a temporary connection between the amniotic cavity and the yolk sac (6). Coordinated degradation of the notochord begins around the fifth week of gestation and mouse models provide evidence that the condensed cells persist into postnatal life and form the nucleus pulposus of the intervertebral discs (27–29). It is important to note that these observations were largely made on animal models such as rodents and birds, mainly chickens, and cannot reliably be extrapolated to humans owing to species differences (30, 31).

### Neurulation

Neurulation not only forms the basis for the largest part of the nervous system, but is also prerequisite for the establishment of the three-dimensional body shape. Neurulation has been studied in detail mainly in chickens-in addition to human and mouse models-many of the insights gained have come from this chicken model (32).

Neurulation comprises two processes, primary and secondary (Figures 2, 3). Before they are initiated, neural induction separates the primary ectoderm into neuroectoderm and surface ectoderm (33). Thereafter, primary neurulation starts when the neural ectodermal layer is stimulated by the notochord to form the neural plate. This process has already been initiated during elongation of the notochord, which acts as an inducer for neural differentiation, in the anterior direction. Primary neurulation is initiated at the neural plate, a condensation of specialized ectodermal cells, and starts around day 19 after fertilization. The plate undergoes fundamental transformations of shape (Figure 2), cell morphology and size, during which the neural plate bends, the prerequisite for forming neural folds (32, 34).Furthermore Chang et al. demonstrated in Brachyury knock out mice that folic acid deficiency interferes neural induction via inactivation of the FGF pathway (35).

Fusion of the neural folds through bending or buckling of them around specifically-defined median and dorsolateral hinge points starts cranially and progresses caudally (32) (Figure 3). The encephalon arises from the most anterior point and the medulla originates in the more caudal parts. In murine models it could be shown that signaling molecules such as PAX3, members of the Zic-family and Cdx2 have a critical influence on primary neurulation (36–40).

Primary neurulation ends with closure of the anterior and posterior neuroporus and is immediately followed by secondary neurulation, which entails epithelization and tubulogenesis of the tail bud (41). The tail bud, an aggregate of undifferentiated, axially-condensed mesodermal cells, is located between the notochord and the primary neural tube in the most caudal spinal area (42, 43). Via a mesenchymal to epithelial transition and cavitation, the neural tube is formed from this solid epithelial cord in the lower sacral and coccygeal regions along the rostro-caudal axis (33, 44, 45). Subsequently, the secondary neural tube becomes continuous with the primary neural tube at the level of somite 27 in chickens, as demonstrated by Le Douarin et al. (46). However, there are insufficient data on the mechanism of secondary neurulation in human embryos. Contrary to other hypotheses, it is now supposed that the tail bud is not a bunch of totipotent cells but is divided into individual territories with defined fates (33).

Meanwhile, the mesodermal layer also undergoes a fundamental change, becoming organized into three parts (Figure 3). The most medial part adjacent to the notochord, the so-called paraxial mesoderm, differentiates into the somites. The intermediate mesoderm, the anlage of the urinary system, is more lateral. The lateral plate mesoderm (LPM) is more lateral still, initially consisting of solid tissue next to the extraembryonic mesoderm. Cavities arise within the LPM during further differentiation. These cavities merge, and two separate mesodermal layers – the parietal and visceral mesoderm - now border the intraembryonic celomic cavity, which is the anlage for the pleural, pericardial, and peritoneal cavities. The parietal pleura, the pericard and the parietal peritoneum arise from the parietal mesoderm - obsolete also called somatopleura, and similarly the visceral pleura, the epicard and the visceral peritoneum arise from the visceral mesoderm – obsolete also called splanchnopleura. Initially the extraembryonic and intraembryonic coelom are connected, however, this connection is subsequently lost due to craniocaudal and lateral folding.

### Somitogenesis

Somitogenesis represents one of the earliest forms of segmentation (47). The early paraxial mesoderm, also called presomitic mesoderm, becomes temporo-spatially organized in a periodic pattern into segmented tissue blocks called somites (Figures 2, 3). Under the influence of oscillating cyclic gene activities such as FGFs, Wnt, BMPs and Notch pathways, and by the mesenchymal to epithelial transition, the paraxial mesoderm begins to take the form of epithelial spheres which could be demonstrated in mice and chicken (48–50). The rhythm of this segmentation process is species-specific, e.g., every two hours in mice and every 90 min in chicken (47, 51–54). This evolutionary mechanism is described as the clock-wavefront model: a “clock” determines the time of differentiation of the somites and the “wavefront” determines the locations of their segmentation (55).

The polarity of the somites in all body planes is established early during embryogenesis. The somites are composed of pseudostratified epithelium (ÓRahilly) surrounding a central cavity called the somitocele (Figures 2, 3). Mature somites consist of the sclerotome, which is the origin of the axial skeleton, and the dermatomyotome, which gives rise to the myotome and the dermatome. The myotome gives rise to the muscles of the back, the thorax, the ventral body wall, and the limbs. The dermatome is the anlage of the dermis of the back (56–58).

Muscles that originate entirely in the somatic, paraxial environment are called primaxial, whereas muscles cells surrounded by the lateral plate mesoderm are called abaxial. The dermatomyotome is divided into hypaxial and epaxial parts: muscles of the ventral body wall originate in the hypaxial part and are innervated by the ventral rami of the spinal nerves. Epaxial muscles are located dorsally from the base of the skull to the tail and are innervated by the dorsal branches of the spinal nerves (59–61).

During the further course of development, the somites lose their epithelial characteristics via epithelial to mesenchymal transition and give rise to the above-mentioned structures (62).

### Yolk Sac

**Figure 4:**
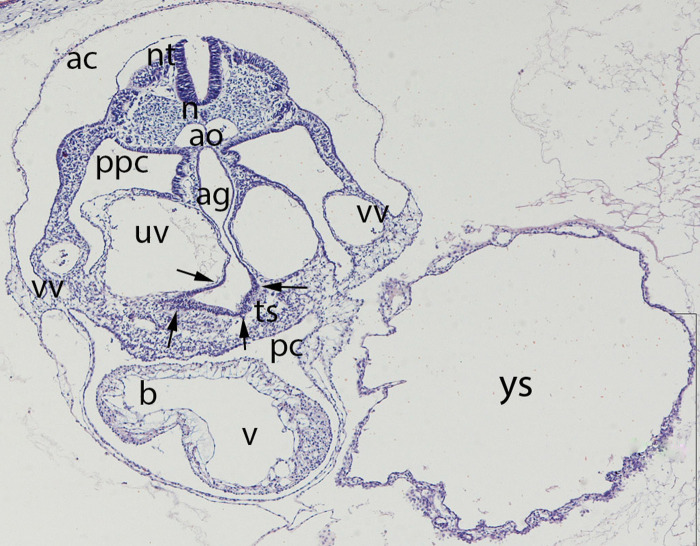
Embryo GV-5 (4 mm). Stage 11 (13–20 somites; 2.5mm-4.5 mm; 24 days). Collection Orts LLorca (Complutense University of Madrid). Photo provided by Prof. J.F. Rodriguez-Vazquez. Axial section of the thoracic segment (4X). Staining. H-E. The section shows the pericardic cavity (pc) surrounding the ventriculus (v) and bulbus (b) cordis. Dorsal of this is the transverse septum (ts). The liver diverticula emerging from the anterior gut (ag) which are growing stringy into the the transverse septum (arrows) are visible. The anterior gut is surrounded by the umbilical (uv) and vitelline veins (vv). pleuroperitoneal cavity (ppc), amniotic cavity (ac), secondary yolk cavity (yc), duplicate aorta (ao), neural tube (nt) and the notochord (n)

The mammalian yolk sac originates in hypoblastic cells from the inner cell mass also known as the primitive endoderm. Mammals generate a transient primary yolk sac followed by a secondary yolk sac, which in humans provides nourishment to the embryo until the end of the first trimester (Figures 2–4). The formation of the extraembryonic mesoderm, which is essential for implementation of the amnion, chorion, and allantois, coincides with establishment of the primary yolk sac. Both the cuboidal visceral endoderm and the parietal endoderm that lines the trophoblast contribute to forming the primary yolk sac, establishment of which is approximately complete at day 12 after fertilization (63, 64). Electron microscopic studies indicate that the extraembryonic mesodermal lining of the yolk sac comprises delaminated cells of the parietal and visceral endoderm (65–67).

The secondary yolk sac forms about day 13 by collapse of the primary yolk sac (Figure 4) and subsequent constriction from it (64, 68). The visceral endoderm now grows strongly while delamination of the parietal endoderm continues.

There are two possible ways for nutrients to reach the embryo: via the vessels in the yolk sac, or via the cavity of the sac, which is the anlage for the future intestine.

### Folding of the Embryo

Concurrently, the superficial and parietal lateral mesoderm grows ventrally to form the lateral body folds. Through the fusion of these folds in the median plane, the typical fetal cylindrical position arises. The parietal layer of the LPM, the surface ectoderm and the amnion become continuous with their equivalents on the opposite side (Figure 4). Similarly, the visceral mesoderm and the endoderm fold in from the lateral side and the endodermal sheet is everted into the inner part of the embryo to form the anlage of the intestinal tube (Figure 4). The right and left body wall are established through this lateral folding, which occurs because of the rapid growth of the somites and the LMP. The intestinal tube remains in contact with the yolk sac via the omphaloenteric or vitelline duct, which is obliterated physiologically during the fifth month of gestation. In the region of this duct, the visceral layer of the lateral plate mesoderm comes into direct contact with the mesoderm of the body stalk with the allantois. The connecting stalk subsequently builds the mesenchymal core of the umbilical cord, which develops around the omphaloenteric duct and allantois. The surface layer of the umbilical cord is built from the amniotic membrane. The main hypothesis regarding the pathogenesis of gastroschisis is inadequate incorporation of the yolk sac during the craniocaudal and mediolateral foldings of the embryo (6, 48, 69). Due to the lack of suitable animal models genetic work-up has become much more difficult here: in mice bone morphogenetic proteins are key players in ventral folding. In knock out mice BMP2 seems to play a significant role in initiation and coordination of lateral folding of embryos. However, here too the findings cannot be transferred one-to-one to humans: Due to the flat shape of the human epiblast - in contrast to the murine cylindrical shape – there is no need in the human embryo for a complete rotation to internalize the intestines (70, 71)

### Establishment of the Body Cavities and First Body Wall Closure

The establishment of the primary body wall is mainly driven by lateral folding of the embryo and is completed around the fifth week. This fact makes distinction between insufficient folding and secondar events, which lead to incomplete body wall closure even more difficult.

Both the ectodermal layer and the somatopleura start to elongate and finally fuse in the midline ventral to the umbilicus. The primary ventral body wall is composed of lateral plate mesoderm and the overlying ectoderm, which are replaced during the further course of development by functional and connective tissue (72). GATA 4 deficient mice exhibit severe defects in the ventral body wall because of disrupted cranio-caudal and ventro-lateral folding (73).

**Figure 5:**
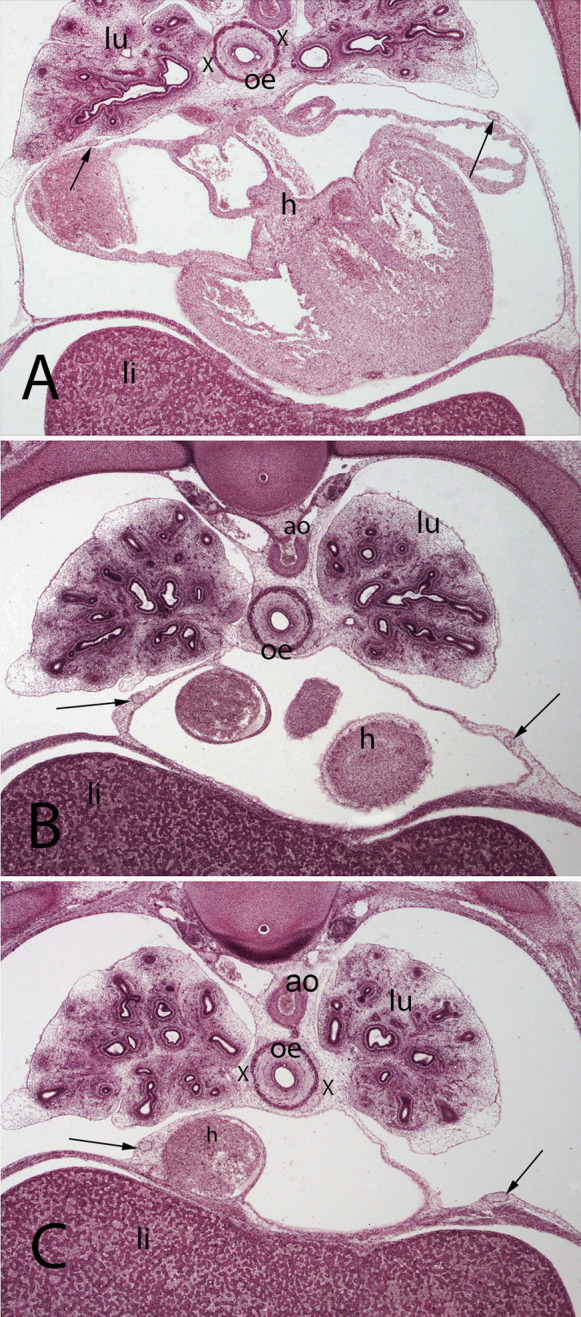
(**A–C**) Embryo A (13 mm). Stage 17 (41 days). Collection Javier Puerta (Complutense University of Madrid). Axial sections of the upper thoracic (**A**), lower toracic (**B**), upper abdominal level (**C**) segments (2X). Staining: H-E. In this sections it is possible to see the course of the phrenic nerves in the pericardial membranes until reach the diaphragm (arrows). Moreover, it is possible to see the aorta (ao) as wells as the esophagus accompanied by the vagus nerves (X). lungs (lu) and heart (h)

Through these folding processes in the course of the fourth week of development, the intraembryonic coelom loses its connection in large segments with the extraembryonic celom. The former subsequently creates a uniform cavity - the coelomic or pleuroperitoneal cavity – which extends from the thorax to the later pelvis. The pericardial cavity which originates from the cranial part of the coelomic cavity surrounds the anlage of the heart. During the folding processes the anlage of the heart as well as the pericardial cavity are translocated in ventro – caudal direction in front of the foregut (Figure 4). The precursor of the pleural cavity – the narrow pleuroperitoneal ducts, are initially located dorsally of the pericardial cavity and provide connection between the pericardial and peritoneal cavity. Around the fifth gestational week the pleuroperitoneal ducts extend into the pleural cavities because of enlargement of the pulmonary buds. Due to further expansion of the pleural cavities two folds appear: cranially the pleuropericardial fold and caudally the pleuroperitoneal fold. In the pleuropericardial fold are the phrenic nerve and the common cardinal vein located (Figure 5). These folds increasingly constrain the connection between the three cavities – the remaining passages are now called pleuropericardial and pleuroperitoneal ducts (69). Due to the continuously growth of the lungs and subsequently the pleural cavities in medio-lateral direction the pleuropericardial membrane develops in the midline between those. This membrane ends dorso - cranially into the pleuropericardial fold (Figure 4). By fusion of the pleuropericardial duct and the pleuropericardial fold at their medial site, a complete separation of the pleural and pericardial cavities occurs in the seventh week.

**Figure 6:**
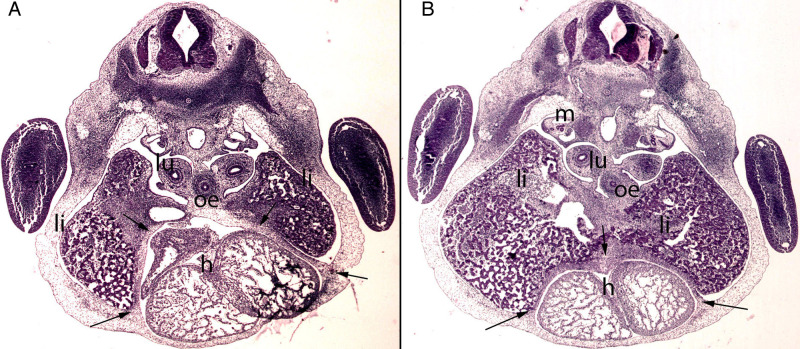
(**A,B**) Embryo BI-8.5 (8.5 mm). Stage 15 (33 days). Collection Jvier Puerta (Complutense University of Madrid). Axial sections of the upper thoracic (**A**) and lower toracic (**B**) segments (2X). Staining: H-E. This sections shows the liver cords (li) growing into the transverse septum (ts) with the consequence increase of the liver size The diaphragm (arrows) is located between the pericardial cavity surrounding the heart and the primitive liver as a dense mesenchymal condensation. The pleuroperitoneal cavity still remains continuous. anterior gut (ag), stomach (s), bulbus (b) and ventriculus (V) cordis, umbilical vein (uv), right primary bronchi (lu), mesonephros (m).

**Figure 7:**
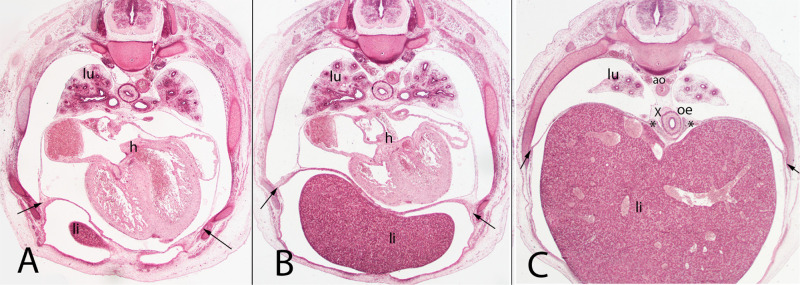
(**A–C**) Embryo A (13 mm). Stage 17 (41 days). Collection Javier Puerta (Complutense University of Madrid). Axial sections of the upper thoracic (**A**), lower thoracic (**B**), upper abdominal (**C**) segments (all 2X). Staining: H-E. liver (li), sternal, costal, lumbar (arrows) and vertebral portions of the diaphragm (*). The pleuroperitoneal cavity still remains continuous. heart (h) aorta (ao), right bronchi (lu).

Through expansion of gaps in the coelom and approximation of them in the midline the peritoneal cavity arises with a small tissue plate in-between, from which the dorsal (and ventral) mesenterium originates. The peritoneal cavity is in contact with the extraembryonic mesoderm via the omphaloenteric duct and loses this connection not until the tenth week of gestation with the return of the intestinal loops into the abdominal cavity. Due to the rapidly progressing growth of the liver the peritoneal cavity also enlarges and the pleuroperitoneal fold now divides as pleuroperitoneal membrane the pleural from the peritoneal cavity (Figure 3). The connection of these two cavities, which is given by the pleuroperitoneal ducts is maintained and remains until the end of the second gestational month (Figures 6, 7) (74).

### Septum Transversum

**Figure 8:**
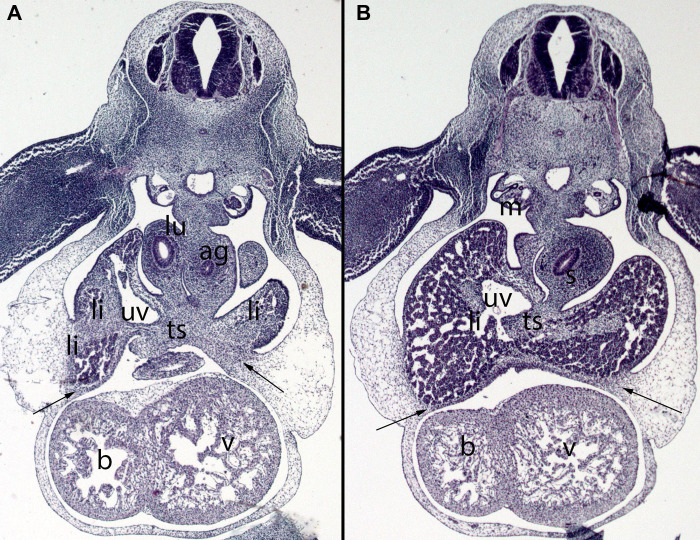
(**A,B**) Embryo DD-10 (10 mm). Stage 16 (37 days). Collection Javier Puerta (Complutense University of Madrid). Axial sections of the thoracic (**A**) and upper abdominal (**B**) segments (2X). Staining: H-E. These sections show the liver cords (li) growing into the transverse septum (ts) with the consequence increase of the liver size. The diaphragm (arrows) is located between the pericardial cavity surrounding the heart and the primitive liver as a dense mesenchymal condensation. The pleuroperitoneal cavity continues to communicate. anterior gut (ag), stomach (s), bulbus (b) and ventriculus (V) cordis, umbilical vein (uv), right bronchi (lu), mesonephros (m).

The transverse septum originates behind the base of the pericardial cavity and the roof of the vitelline duct, bordered ventrally by the extraembryonic celom and dorsally by the back region of the embryo (Figure 4). It consists of mesenchymal tissue and, as the name implies, is a separating wall in the transverse plane. However, it does not completely separate the thorax from the abdomen, since the pleuroperitoneal ducts on both sides are continuous between the two cavities. In the early organogenesis the endodermal liver primordium is embedded into the mesenchyme of the transverse septum and is rapidly gaining in size to account for about 10% of the body weight in the ninth week (75) (Figures 6–8). Subsequent studies indicated that with further differentiation the transverse septum is incorporated as the centrum tendineum of the diaphragm, though to date there have been no lineage studies of its fate (76, 77).

### Diaphragm

The diaphragm is composed of the transverse septum, the pleuroperitoneal folds, the dorsal mesentery of the esophagus and parts of the body wall (78, 79). It is divided into the costal part, which has respiratory and barrier functions, and the crural part (Figure 7). The costal part has radially arranged myofibers extending from the ribs to the central tendon with interposed connective tissue (80, 81). Organogenesis of the diaphragm is completed by week 12 of gestation. Its correct muscularization requires a precisely choreographed sequence of molecular pathways that enables the somitic precursor cells to delaminate, migrate and invade the diaphragmatic anlagen (82).

Studies of knockout mice lacking diaphragm differentiation have revealed that c-met and Pax3/7 are crucial for the migration of muscular progenitor cells into the diaphragm muscle (79, 83–85). These progenitor cells originate in cervical somites 3–5 and migrate during embryogenesis into the pleuroperitoneal membrane/folds.

The terminology has become confused: some authors have also described a posthepatic mesenchymal plate (PHMP) (76, 86, 87). This structure is located dorsal to the liver and ventral to the pleuroperitoneal canal, grows in the dorsolateral direction and closes the pleuroperitoneal canals to separate the thoracic from the abdominal cavities. Iritani reported that incomplete differentiation and growth of the PHMP with subsequent failure of fusion with the pleuroperitoneal folds leads to diaphragmatic defects (87). Morphological investigations of mice using scanning electron microscopy showed that the PHPM or its malformation plays a significant role in the formation of a CDH. (76).

**Figure 9:**
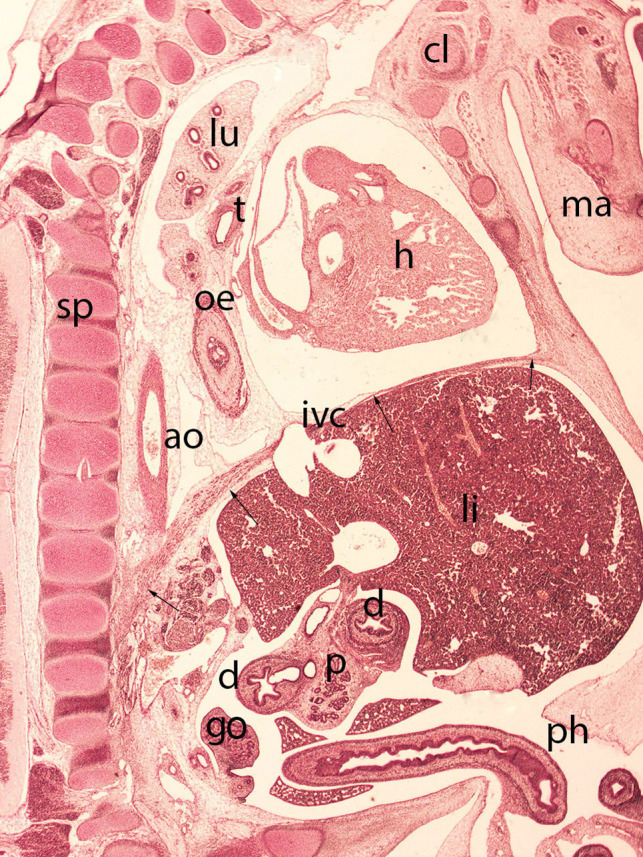
Embryo HA-24 (24 mm). Stage 22 (54 days). Collection Javier Puerta (Complutense University of Madrid). Sagittal section of the trunk (1X). Staining: Picro. The descensus of the diaphragm and its typical dome shape, with the anterior sternal and posterior vertebral attachments is shown (arrows). The hole in the phrenic center is visible with the inferior vena cava (ivc) and behind these structures the descensus of the esophagus (oe) and aorta (ao) for passing through their corresponding hiatus. Clavicle (cl), mandible (ma), spine (sp), duodenum (d), pancreas (p), gonad (g), liver (li), heart (h) lung (lu), trachea (t) and physiological hernia (ph)

The pleuroperitoneal folds are described as pyramid-shaped structures that form transiently between the pleural and abdominal cavities (Figure 9). According to knockout studies, they emerge from the lateral plate mesoderm (88, 89). Subsequently, they extend medially and ventrally and give rise to non–myogenic diaphragmatic connective tissue. Merrell et al., studying mice carrying Prx1–cre (90), showed that the pleuroperitoneal folds regulate diaphragmatic muscle development and are also the source of the central tendon (91). Interestingly, studies of mouse embryos showed that the nascent diaphragm develops at the cervical level and migrates during further development to the base of the thoracic cavity (89). However, despite the agreement among embryology textbooks about the organogenesis of the diaphragm, many of the concepts are based on a single publication by Wells, which was a morphological postmortem study of human embryos (92).

**Figure 10:**
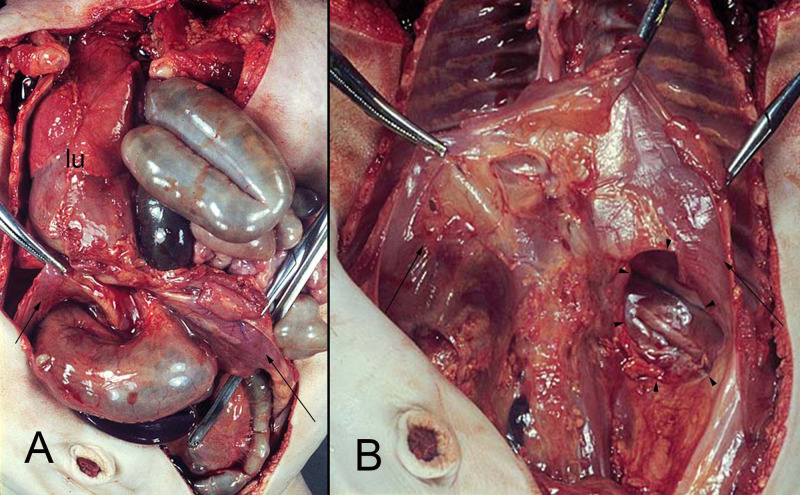
(**A,B**) hernia of the intestine into the left pleural cavity (Bodaleck) of a neonate. After removing the intestinal contents the diaphragm (arrows) the permanent defect of the pleuroperitoneal membrane (arrowheads) is visible. The left lung (lu) has been displaced leftwards.

The most widely accepted hypothesis is that congenital diaphragmatic hernias are caused by morphogenetic defects in the pleuroperitoneal folds (Figure 10). In consequence, proper establishment of the costal muscle and its surrounding connective tissue fails and deficiencies in the diaphragmatic barrier result, with subsequent herniations of abdominal tissue (82, 93, 94). Various genes potentially responsible for proper diaphragm development have been identified in human embryos affected by CDH, for example GATA 4, c-Met, Wt1, and COUP-TFII (77, 95–101), but whether every genetic defect result in a corresponding phenotype in humans is not yet clear.

Another hypothesis about the pathogenesis of CDH is that the pleuroperitoneal canals remain open, enabling the abdominal organs to herniate (92, 102, 103). However, CDH results from early failure of differentiation of the pleuroperitoneal folds (94, 97). Impaired lung development and subsequent alteration of the developing PHMP was also suspected of being responsible for CDHs. However, studies with FGF10–knockout mice disproved this hypothesis; mice with absent lung development showed inconspicious organogensis of the diaphragm (97, 104).

Furthermore, morphogenesis of the diaphragm is tightly connected to the phrenic nerve, and vasculogenesis and morphogenesis of the costal muscles (89). Appropriate axonal outgrowth, targeting of the myoceptors and branching of the phrenic nerve are crucial for the proper development and function of the diaphragm, but it remains to be elucidated whether CDHs result from phrenic misgrowth, or whether the malformation and mis-targeting is directly caused by CDHs (Figure 5) (94, 105).

Most hernias of the diaphragm occur on its left side (106) (Figure 10). According to some authors, this mainly left-sided occurrence of CDHs could be attributed to the bigger contact area on the right with the major part of the liver. The contact area on the left side is smaller, so the diaphragm needs longer for its closure (76).

### Ribs

From an evolutionary point of view, the ribs evolved because the stabilizing exoskeleton was lost (107, 108). The proximal and distal parts of the ribs originate in the medial and lateral sclerotomes of somites, and their outgrowth is coordinated by conserved HOX-gene expression (109–113). The sternal parts of the ribs are of abaxial origin, as demonstrated by experiments in which somatic cells were mechanically prevented from invading the somatopleura; the vertebral parts of the ribs developed adequately but the sternal parts were missing (114–117) (Figures 7, 9). Furthermore, Wood et al. (113) showed that for proper rib development, beyond the myogenic regulator gene family, sufficient muscle organogenesis and muscle interactions are required. Anatomically and functionally, the rib cage is divided into upper and lower parts. The first to seventh ribs of the upper section are referred to “true” ribs and are attached to the articular surfaces of the sternum. The eighth to twelfth ribs of the lower part are called floating or “false” ribs because they have no articular connection to the sternum. Differentiation and development of the rib cage differ between these parts in signaling and rates, but the two sections are interdependent, induced and influenced by each other (118).

**Figure 11:**
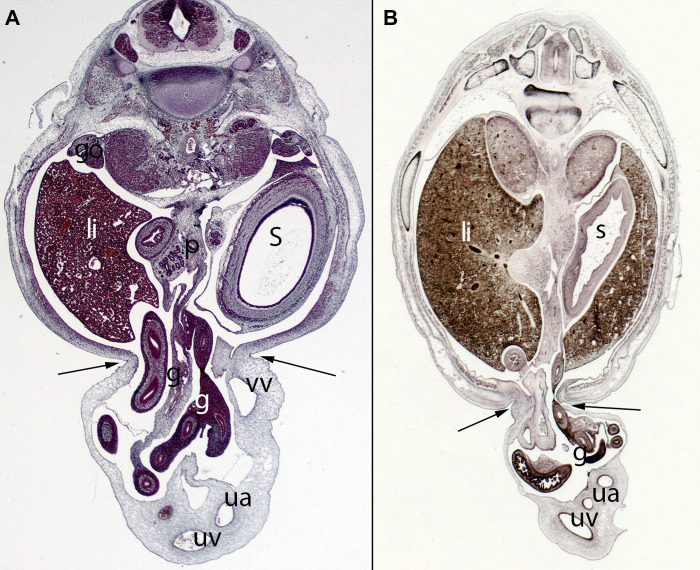
(**A**) Embryo ES20 (20), (Stage 20, 51 days), axial section (1X), Staining: H-EF. (**B**) Embryo VD-34 (34 mm), (57 days), axial section (1X), Staining: Bielschowsky, Collection Javier Puerta (Complutense University of Madrid). Physiological hernia and its narrowing during the embryonic period (arrows). In these sections, you can see the loops of intestine in the umbilicus and the narrowing umbilical orifice. gut (g), stomach (s), liver (li), umbilical vein (uv), umbilical artery (ua), gonad (go), pancreas (p), vitelline vein (vv)

**Figure 12:**
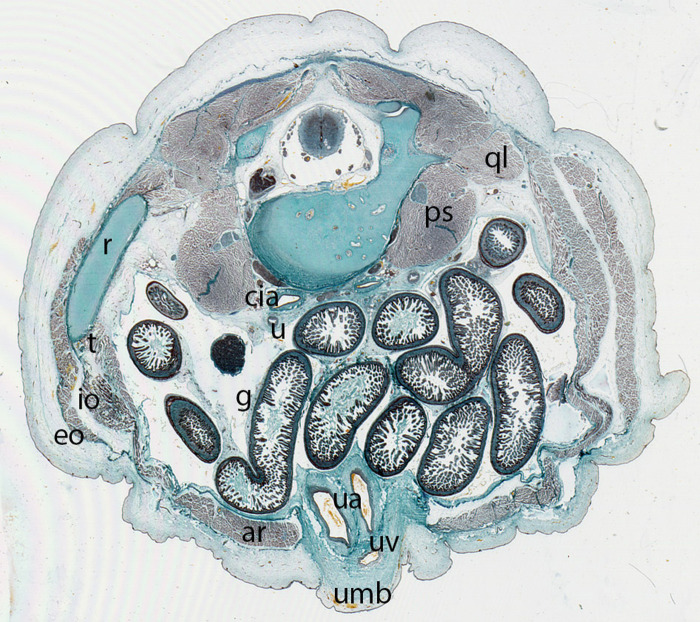
Fetus F88 (88 mm), (12th weeks). Collection Javier Puerta (Complutense University of Madrid). Axial section of the trunk (1X). Staining: Azan.In this section the umbilicus (umb) and the anterolateral abdominal muscles: rectus abdominis (ar), external oblique (eo), internal oblique (io) and transversus (t) abdominal muscles are visible. M. psoas (ps) and M. quadratus lumbarum (ql). common iliac arteries (cia), ureter (u), intestinal loops (g) and the contents of the umbilicus, the umbilical arteries (ua) and vein (uv)

The ribs are also subject to resegmentation; caput, collum and the costal tubercles arise from the cranially located somite, while the ventrally-located corpus originates from the caudally-located somite. During embryogenesis the ribs are cartilaginous; they ossify during the fetal period (Figures 9, 11B, 12).

### Sternum

As a component of the appendicular skeleton, the sternum was believed to emerge from the distal ends of the ribs (Figure 9). However, explant studies have revealed that the paired sternal anlagen originate in the lateral plate mesoderm located in the lateral somitic frontier (110, 117, 119–123). The sternal anlagen on the two sides migrate towards the midline and finally merge there. In a fate map study, Bickely and Logan demonstrated the crucial role of Tbx5 in sternal development, as also apparent in Holt–Oram syndrome, which is characterized by sternal defects (119).

### Umbilical Cord and its Vessels

The prerequisite for forming an umbilical cord is the connecting stalk, which secures the embryo in the chorionic cavity during the third week. Because of cranial-caudal folding, the connecting stalk approximates the yolk sac. From this approximation emerges the rudimentary umbilical ring, which is not covered by the rapidly-growing amnion (124). The umbilical cord forms between the fourth and sixth weeks when the vitelline duct and the connecting stalk become wrapped in the amnion sheet; it contains the two umbilical arteries and one umbilical vein surrounded by Wharton’s jelly. The outer wrapping of the umbilical cord is formed by the amnion. During the sixth week of gestation, the developing intestines herniate via the umbilical ring into the tissue of the cord to enable adequate gut rotation and repositioning (Figure 10). The intestinal loop returns into the embryonic body at about the tenth week after fertilization (Figure 12). The umbilical ring is the transition between amnion and ectoderm.

### Physiological Umbilical Herniation

Herniation of the intestine into the extraembryonic celom begins around week six after fertilization. The process is called “physiological umbilical herniation” and is due to the rapid elongation of the intestinal tube. It is accompanied by a 90° clockwise rotation of the intestinal loop (125). The return from the celom back into the intraembryonic cavity is again connected with a counterclockwise rotation of 180°. These rotations ensure the correct positioning of the intestine (Figures 11, 12).

The return of the intestinal loops is the starting signal for (secondary) closure of the ventral body wall, but the order of return is not without controversy. Some authors state that the cecum is the last segment to return (126, 127); others report that the distal ileum is the final returning segment (128–130). Studies of the return of the intestine loop into the embryonic celom in human embryos/fetuses are rare (128, 131, 132). Mall described the intestine as being “sucked back” into the peritoneal cavity because of the rapid growth of the latter (133). Another hypothesis proposed by Frazer and Robbins (127) states that the intestine returns into the abdominal cavity in a coordinated manner, proximally to distally, each segment slipping back because of retraction forces; the so-called rope model. According to the authors, this orderly return of the intestine is driven by a narrow umbilical orifice and the “amniotic pressure on the umbilical sac” (127). Soffers et al. (132) support this model of orderly withdrawal in a proximal to distal direction, additionally describing secondary and tertiary coil formation.

### Second Body Wall Closure

The prerequisite for the second body wall closure is ventral migration of the somitic cells.

Muscles in which the somitic cells originate entirely from one somite or dermatomyotome are called primaxial (116), whereas somitic cells that originate in the somitic frontiers, the border area between the somite and the lateral plate mesoderm, are termed abaxial. Anatomically, however, they are classified according to the nerves involved: Ventral hypaxial and dorsal epaxial. The hypaxial muscles, which are accompanied by rami ventrales, are responsible for ventral body wall closure. The rectus abdominis and the external and internal oblique abdominis muscles develop before the return of the intestinal loop into the abdominal cavity (Figure 12) (59). Therefore, these muscles have not yet assumed their original positions, and the rectus muscles clearly show a diastasis in the midline (59, 134).

### Formation of the Inguinal Canal

**Figure 13:**
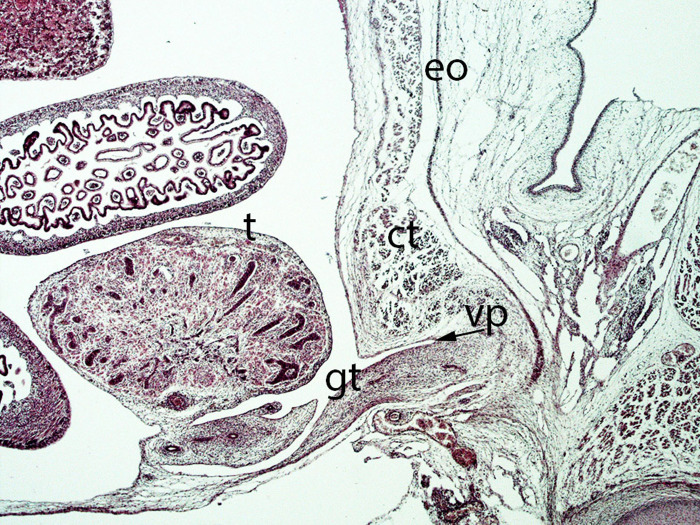
Fetus F25 (60 mm), (10th weeks). Collection Javier Puerta (Complutense University of Madrid). Sagittal section of lower third of the abdominal cavity (2X). Staining: Azan. The gubernaculum testis (gt) inside the inguinal canal and surrounded by the vaginal process (vp) is visible. The testicle (t) is located dorsally of the internal ring of the inguinal canal. external oblique (eo) muscle, conjoint tendon (ct).

**Figure 14:**
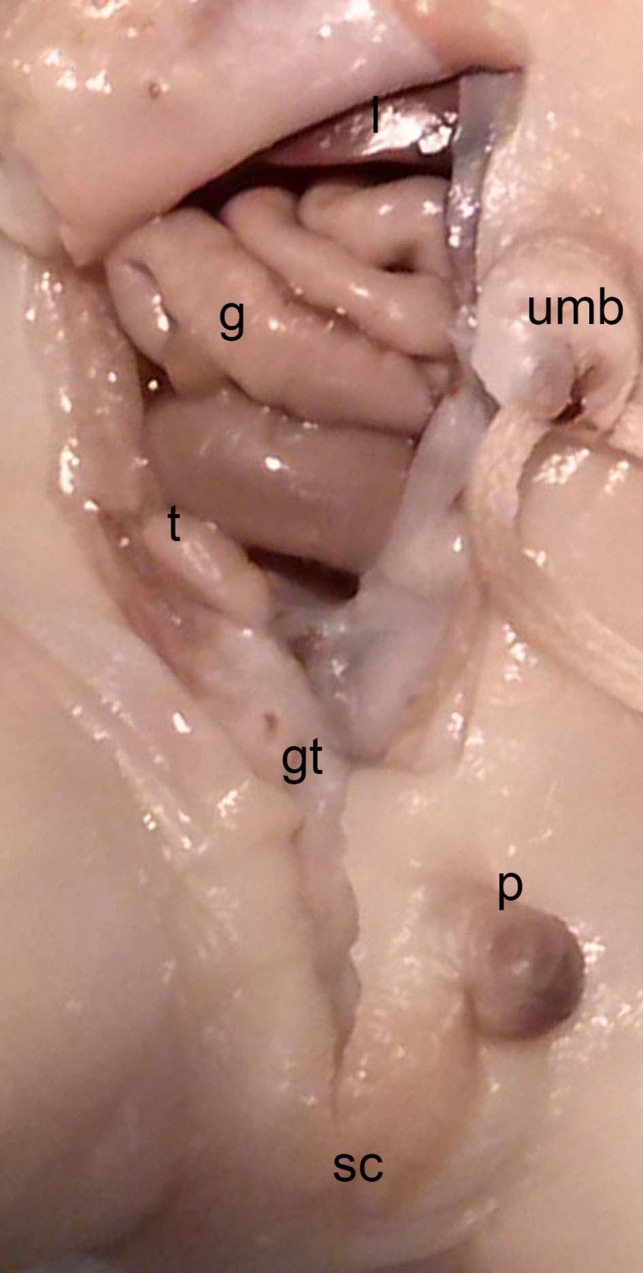
Dissection of the lower abdominal wall and scrotum in a male neonate (BOU; 30.2 cm; 28–32 weeks) with the testicle (t) inside the peritoneal cavity located dorsally of the internal inguinal ring and the Gubernaculum testis (gt) as an amorph substance; sc, scrotum; intestine (g), p, penis; umb, umbilicus.

**Figure 15:**
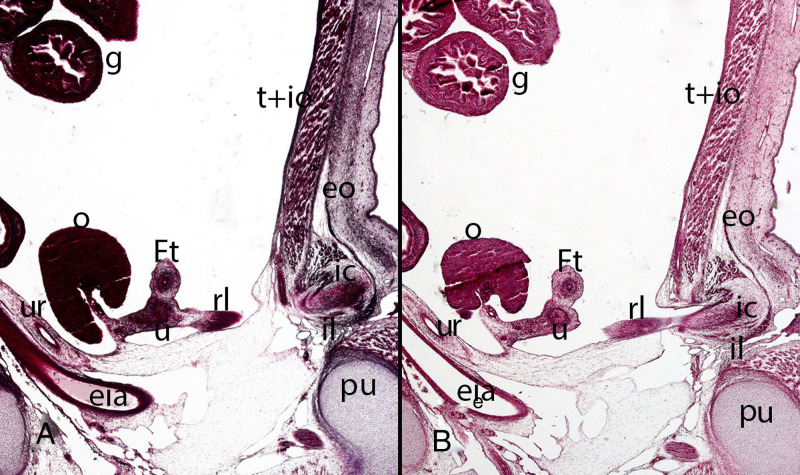
Fetus F14 (48 mm). (9th weeks). Collection Javier Puerta (Complutense University of Madrid). Sagittal sections (**A** and **B**) of the trunk (1X). Staining: Azan. The inguinal canal (ic), inguinal ligament (il), the external oblique muscle (eo) and the transverse (t) and internal oblique muscles (io) are visible. Inside the inguinal canal the gubernaculum testis is located which has become in this case of a female specimen the round ligament (rl) connected with the uterus (u). ovary (o), Fallopian tube (Ft), ureter (ur), external iliac artery (eia), pubis bone (pu), intestinal loops (g) in the peritoneal cavity.

The dynamic development of the inguinal canal (IC) is closely connected to differentiation of the gonads and their migration into the extracorporal scrotum (Figures 13, 14). There have been debates as to whether the formation of the IC underpins a defect in the descending gonads, or whether the defect is preformed and already present when gonadal migration starts (135). In favor of the latter view, the IC is established in both sexes even though descensus testis only occurs until the scrotum in the male (Figures 13, 14), whereas in the female the ovary remains inside the pelvis because of the Fallopian tube whose presence act as a barrier. This in further consequence in female the gubernaculum testis became the round ligament that reaches the mons of venus (Figure 15). In several morphological studies, the confines of the IC were already identifiable from the tenth week onwards (136, 137), as various researchers have proposed (138–141). The question also arises as to whether the formation of the IC is oriented to the growth and direction of the ilioinguinal nerve.

### Descensus Testis

As the testis differentiates intraabdominally, its descent must be through the IC guided by the gubernaculum testis (GT) (Figure 13). The gubernaculum originates in two triangular mesenchymal condensations, the inguinal plica dorsal to the Wolffian duct and the inguinal crista opposite the plica (141). The growth and differentiation of the GT is induced by insulin-like 3, which secreted by Leydig cells (142, 143). The pelvic portion of the GT is created by merging the mesonephric and inguinal portions. The inguinal portion continues to extend as far as the superficial inguinal ring, which is formed by the external oblique muscle. Condensation of the mesenchyme progresses caudally to form the scrotal part of the ligament. Notably, the GT is not connected to the gonads at any time during embryogenesis. At the end of this period the vaginal process appears, an protrusion and evagination of the peritoneum that surrounds the pelvic and inguinal portion of the GT (144). The GT and the processus vaginalis herniate together through the abdominal wall accompanied by the transversalis fascia and the internal and external oblique abdominal muscles. During further development, the vaginal process elongates and eventually envelops the GT. At the end of the fetal period the process has traversed the full length of the IC and reached the scrotal sac. The IC acquires its adult morphology with anterior and posterior ligaments as well as the oblique course during the fetal period. This is due to the continuous growth of the abdominal muscles and wall, with the accompanying displacement of the inguinal rings (Figure 14). During their growth and differentiation, the testes come to lie on the anterior abdominal wall close to the inner inguinal ring. It is controversial whether the GT acts to guide the testes through the IC, or actively pulls them into the scrotum. In contrast to the development of the GT in male specimen, the GT in female is remodeled into the round ligament in female fetuses. Here the vaginal process only reaches the initial section of the IC and the ovaries do not reach the abdominal wall (Figure 15) (141).

## Final Remarks

For visceral surgeons who deal with congenital abdominal malformations it is mandatory to be familiar with the different pathological entities. This is not only important for diagnosis but also for further treatment and therapies. It makes a huge difference whether if it is for example a in most cases self-limiting IUH, which needs consequent observation or a life-threatening condition like gastroschisis. For finding the correct diagnosis it is necessary to be familiar with the physiological as well as pathophysiological embryogenesis. The heterogeneity of disease patterns and varieties resulting from dis – and interruption of developmental process underlines the importance of embryology for clinicians starting with the diagnosis up to the therapy. Furthermore, knowledge about of the relationship between possibly asymptomatic variants and potentially resulting pathologies enables surgeons to grant patients an adequate treatment. Keeping that in mind several of the following malformations of the abdominal wall may encounter surgeons in their professional career: Gastroschisis is defined as congenital, structural defect of the abdominal wall with a protrusion of the viscera through it. The eviscerated intestine is not covered by the amnion und thus is directly exposed to the amniotic fluid (145). Its etiology is controversial discussed: some studies emphasize the role of genetic factors and an increased familial risk (146–149), whereas other research work did not support an increased familial risk (150–152). In recent decades, several causes for the development of gastroschisis have been discussed: failure of mesodermal formation (12), rupture of the amnion around or beside the umbilical ring (153), thrombosis of the umbilical vein caused by estrogen (154), malformation of the right vitelline artery (155), defective invagination of the secondary yolk sac and omphalomesenteric duct – nevertheless with regular abdominal wall formation (156). A significant breakthrough in the study of gastroschisis was achieved in 2013 by Rittler and colleagues (157). They described gastroschisis as a defect of the umbilical ring in five stillborn neonates: the umbilical cord was “only attached to the left side of the umbilical ring, while the right side remained uncovered allowing evisceration”. This account was corroborated by Bargy and Beaudoin (158), who investigated 121 embryos and fetuses and proposed that gastroschisis is due to “amniotic rupture along the umbilical cord in its pars flaccida between weeks 8 and 11 of gestation”. They showed that amniotic continuity, which is established around day seven of gestation, is missing at the right side of the umbilical cord in fetuses affected by gastroschisis, with consecutive herniation of the midgut and the ascending colon. Furthermore, they found that the peritoneal recess was open on the right side of the umbilical cord, the right side of the umbilical vein being covered by peritoneum but the left side being “normal”. This right side dominance in the defect of the amnion could, according to the authors, be due to the predominance of the left umbilical vein.

Congenital diaphragmatic hernias occur in 1 in 25000 live births and are life-threatening malformations with a mortality rate of about 50%. Out of this total number of cases, only 5%–10% have a chromosomal abnormality (159–162). Each hernia is named according to its location: posterolateral hernias are referred to as Bochdalek and anterior-medial ones as Morgagni–Larrey hernias. Bochdalek hernias are the most common, with a prevalence of 70%–75%. Interestingly, the defect occurs most frequently in the left postero-lateral diaphragm. Disruptors of normal diaphragmatic organogenesis such as Nitrofen and the teratogenic effect of vitamin A deficiency are known from experimental animal studies (163, 164). In humans, weeks 4–6 of gestation seem to be critical for diaphragmatic malformations, which occur mainly on the left side of the diaphragm, for still unexplained reasons.

Omphaloceles arise from the persistence of physiological midgut herniation: the intestine that has been translocated into the umbilical cord remains in the umbilical cord and does not return to the abdominal cavity, which leads subsequently leads to malrotation and mispositioning of the intestine. This malformation is more common in children of both older and younger primiparous mothers and also more often affects male children (165). In contrast to gastroschisis, omphaloceles are associated with such syndromes as trisomy 13, 18 and 21, Beckwith-Wiedemann syndrome, Carpenter syndrome and others (166–170).

To be distinguished from omphaloceles are the congenital, infantile umbilical hernias (IUH), which are associated with low birth weight, meconium peritonitis and prematurity (171). These hernias are very common in children with a prevalence of up to 23% in neonates (172). In African cultures IUHs, which are always covered with skin are thought to be a sign of fertility and beauty (173). In most cases, they have no pathological value and often close spontaneously (174). Densler (1977) postulated that umbilical hernias are the result of incomplete closure of the fascia of the umbilical ring (175). More precisely the umbilical vessels failed to fuse with the urachus and the margins of the umbilical skin (171). This results in a persistent connection between the intraabdominal cavity and the extraembryonal mesoderm. Spontaneous closure of the IUHs up to the age of five years are possible (176). However, there is a risk of incarceration of the IUH which is an absolute indication for surgical repair.

A patent processus vaginalis (PPV) is due to an absent or incomplete obliteration of the processus vaginalis. In male the processus vaginalis is a funnel-shaped protrusion of the peritoneum into the scrotum and arises from the denscensus testis. Under physiological conditions, this obliterates after the descent of the testis is complete. However, if it remains open, there is an open connection between the abdominal cavity and the scrotum. In women, the processus vaginalis is also called Nuck's canal. This refers to the peritoneal fold that runs with the Ligamentum teres uteri through the IC to the labia majora. Similar to men, this normally obliterates postnatally.

PPV are protrusions of the peritoneal cavity into the scrotum and labia majora respectively and are the leading risk factors for development of indirect inguinal hernias (IIG) and hydroceles (177). These inguinal, indirect hernias pass through the internal aperture of the inguinal canal and follow the course of the spermatic cord (178). However PVVs do not represent pathology on their own and may remain asymptomatic and may disappear with increasing growth (179). Although the processus vaginalis may occur prenatally studies have evaluated an incidence for PPVs in children of about 60% at the age of seven months (144, 180, 181). Presence of a PPV implies a four times higher risk of developing an inguinal hernia within 5.3 years (182).

In surgical history postnatal surgical intervention serves as a life-sustaining or at least a life-improving measure in most of these malformations. But another point that will increase significantly in importance is early intrauterine repair. Fetoscopic surgery is a rapidly developing field that benefits greatly from the technical advances of the last decades and goes hand in hand with developments in medical imaging. The postnatal morbidity rate in severe cases of lung hypoplasia caused by CDH is similar to the postnatal survival rate of patients with moderate lung hypoplasia after successful intrauterine intervention. However, fetal surgery focuses on pathophysiological conditions such as pulmonary hyperplasia, not on anatomical conditions (183, 184).

Where there is light there is also shadow, and there are problems with fetoscopic surgery: limited numbers of patients, and lack of randomized trials. Therefore, many of the procedures are still considered experimental. At this stage, most procedures performed are directly life-sustaining for the fetus. It should not be forgotten that the mother is also exposed to risk during fetoscopy.

Taking the rising prevalence of abdominal cavity and wall defects into account, the possibilities now opening regarding surgical care and extended genetic clarification offer new approaches to therapy. However, every path is only as good as its foundation, which in this case is a solid understanding of embryology.

The heterogeneity of disease patterns and varieties resulting from dis – and interruption of developmental process underlines the importance of embryology for clinicians starting with the diagnosis up to the therapy. Therefore, this review gives a comprehensive overview of the histological and molecular processes during embryogenesis that are crucial for the normal development of the abdominal wall and the two body cavities. We focus first on gastrulation, the starting signal for the development of the trilaminar germ disc, followed by the organogenesis of specific structures in the abdominal wall.
